# Synovial fluid potentiates local fibroblasts to drive monocyte activation in juvenile idiopathic arthritis

**DOI:** 10.1186/s13075-026-03776-z

**Published:** 2026-02-28

**Authors:** Tobias Schmidt, Anki Mossberg, Petra Król, Meliha C Kapetanovic, Jon T Einarsson, Adam P Croft, Anders A Bengtsson, Fredrik Kahn, Robin Kahn

**Affiliations:** 1 Department of Pediatrics, Clinical Sciences Lund, Lund University, Lund , Sweden; 2 Department of Rheumatology, Clinical Sciences Lund, Lund University, Lund, Sweden; 3 Wallenberg Center for Molecular Medicine, Lund University, Lund, Sweden; 4 Department of Pediatrics, Clinical Sciences Lund, Lund University, Skane University Hospital, Lund, Sweden; 5 Department of Rheumatology, Clinical Sciences Lund, Lund University, Skane University Hospital, Lund, Sweden; 6 Department of Inflammation and Ageing, University of Birmingham, Birmingham, B15 2TT UK; 7 Research into Inflammatory Arthritis Centre Versus Arthritis (RACE), University of Birmingham, Birmingham, B15 2TT UK; 8 National Institute for Health and Care Research (NIHR) Birmingham Biomedical Research Centre, Birmingham, B15 2TH UK; 9 Department of Infectious disease, Clinical Sciences Lund, Lund University, Skane University Hospital, Lund, Sweden

**Keywords:** Synovial fibroblasts, Monocytes, Juvenile idiopathic arthritis, Inflammation, Synovial fluid

## Abstract

**Introduction:**

Synovial fibroblasts (S-Fib) are recognized as key drivers of joint inflammation in adult arthritis. Moreover, data are limited on the processes driving monocyte activation in oligoarticular juvenile idiopathic arthritis (oJIA). Therefore, we aimed to explore if S-Fib from patients with oJIA induce monocyte activation.

**Methods:**

S-Fib were isolated from the synovial fluid (SF) of *n* = 13 patients with oligoarticular juvenile idiopathic arthritis (oJIA) and *n* = 4 patients with rheumatoid arthritis (RA) to be used as a disease control. Healthy adult S-Fib were purchased. The interaction between monocytes and S-Fib was studied in co-cultures with monocytes isolated from healthy donors. These cells were analyzed by surface marker expression, cytokine production and T-cell activation. Moreover, S-Fib were “re-introduced” to the inflammatory synovial environment by priming or not with pooled cell-free SF from oJIA patients. The S-Fib were subsequently analyzed by cytokine production, ability to induce immune cell chemotaxis and mass spectrometry for proteomic changes, as well as their ability to induce monocyte activation as above.

**Results:**

Co-culture with S-Fib induced inflammatory activation of healthy monocytes as characterized by increased CD86 and HLA-DR expression, cytokine production and subsequent T-cell activation. Importantly, there were only minor differences in monocyte activation pattern depending on the source of the co-cultured S-Fib (oJIA, RA or healthy). Interestingly, compared to S-Fib alone, priming of S-Fib with a pool of SF induced an inflammatory phenotype, production of IL-6 and enhanced chemotaxis of monocytes. Additionally, primed S-Fib induced a more pronounced inflammatory phenotype in healthy monocytes compared to S-Fib alone. Finally, direct cell-cell contact between S-Fib and monocytes was required for full induction of the observed monocyte activation.

**Conclusions:**

Our data show a contact dependent role for S-Fib in driving inflammation in oJIA by inducing pro-inflammatory monocytes, processes potentiated by inflamed SF. These data further support that targeting cell-cell interactions could be a viable option to explore for novel treatment strategies in arthritis.

**Supplementary Information:**

The online version contains supplementary material available at 10.1186/s13075-026-03776-z.

## Introduction

Juvenile idiopathic arthritis (JIA) is an umbrella term for the most common types of childhood-onset arthritis [1]. There are seven subgroups, which differ to some extent in clinical presentation and disease course [1, 2]. Oligoarticular JIA (oJIA) is the most prevalent subgroup in the western world, representing roughly 30–60% of all patients with JIA [1, 3]. Both the pathogenesis and etiology of oJIA are largely unknown, but the role of innate immunity, as well as local synovial fibroblasts (S-Fib), in driving synovial inflammation is becoming increasingly acknowledged. We and others have previously shown that synovial monocytes from oJIA patients are activated [4, 5]. Monocytes are believed to contribute to the pathogenesis by impaired clearance, cytokine production and an enhanced ability to promote T-cell activation [5, 6]. However, data on the role of the local cells, S-Fib, in driving immune cell infiltration and activation, is more limited.

The role of S-Fib appears prominent in adult diseases. In rheumatoid arthritis (RA) they are described as inflammatory, destructive and a prominent source of cytokines such as IL-6, IL-8, GM-CSF, and VEGF, which have roles in immune cell infiltration, differentiation of macrophages and in angiogenesis [7]. Different subtypes of S-Fib in the synovial membrane have been described, were the sub-lining S-Fib are of a pro-inflammatory nature, and the lining S-Fib are less inflammatory but more destructive [8]. Furthermore, S-Fib have been identified in the synovial fluid, but less is known about their origin and function, although they seem to be inflammatory [9]. Interestingly, repeated priming of S-Fib induced a more potent phenotype with increased migration and invasiveness in an animal model of arthritis [10]. Moreover, several cytokines found in inflamed synovial fluid drive S-Fib activation [11]. Unsurprisingly, these data suggest that the inflammatory environment may influence the S-Fib phenotype and function.

In contrast to RA, less is known of the role of S-Fib in JIA [12]. The expression of several surface markers found in S-Fib from RA patients have also been identified on S-Fib from JIA patients, and a recent study has comprehensively characterized JIA S-Fib using spatial transcriptomics, highlighting a similar architecture as adult arthritic diseases [12, 13]. Yet, differences in cell populations exist, likely due to factors such as age, which underline the need for pediatric studies. Indeed, at the phenotype level, S-Fib in children display a hypertrophic chondrocyte-like phenotype and are believed to contribute to bony overgrowth and may thus not be as destructive as S-Fib in adult diseases [14, 15]. Indeed, they do not seem to be major sources of MMPs [16]. However, similar to S-Fib in adults, S-Fib in children are known to produce several cytokines and chemokines, such as IL-6, and a recent study found that synovial fluid stimulation results in cytokine production by S-Fib [17, 18].

There is limited knowledge in general on the ability of S-Fib to drive activation of immune cells, specifically myeloid cells such as monocytes. Co-culture between S-Fib (both healthy and arthritic) and monocytes results in increased pro-inflammatory cytokine production, and S-Fib conditioned medium increases viability of monocytes [11, 19]. Indeed, we have previously shown that adult healthy S-Fib promote the induction of inflammatory monocytes [5]. In turn, monocytes and macrophages produce cytokines, such as TNF, that modulate S-Fib function [20].

Thus, few studies have investigated the ability of S-Fib from oJIA patients to induce monocyte activation. Here, we set out using an exploratory approach to investigate if S-Fib derived from oJIA patients induce activation in monocytes, and the effect of prior priming of the S-Fib with inflammatory synovial fluid. By identifying the process of activation, we could potentially elucidate novel mechanisms that could be targeted in future therapies.

## Materials and methods

### Patient material and clinical characteristics

Consecutive patients (*n* = 23) with oligoarticular JIA (oJIA) according to the International League of Associations for Rheumatology (ILAR) at the Department of Pediatric Rheumatology, Skåne University Hospital, Sweden, between 2016 and 2025, were included in this study when undergoing therapeutic joint aspiration. Synovial fibroblasts (S-Fib) were isolated from the synovial fluid (SF) of *n* = 13 patients, and the SF used for priming experiments was pooled in equal proportions from *n* = 8–10 patients with oJIA, depending on material availability. SF used for pooling were from patients who were either untreated or had only received NSAIDs. Moreover, S-Fib were isolated from the SF of *n* = 4 patients with RA in a similar manner as the oJIA patients. The isolation of cells and SF is described below. Clinical characteristics of the patients included in this study are described in Table 1. S-Fib and SF samples were used as available at the time of the experiments, due to factors such as culture status, passages and volume (for SF). To maximize standardization and limit data drift or variation over time, experiments were performed in parallel, and monocyte donors were included within the shortest possible time window for a given experiment.

**Table 1:** Patient characteristics. Descriptive clinical and laboratory data of the oJIA (*n* = 23) and RA (*n* = 4) patients from whom synovial fibroblasts and/or synovial fluid were collected from. From two oJIA patients, we collected both synovial fluid and synovial fibroblasts

Variables	S-Fib	SF	RA S-Fib
Samples, n	13	12	4
Disease duration^1^, median [IQR]	10 [2-63]	21.5 [4.5-104.2]	2.5 [0-7.5]
Age (years), median [IQR]	8 [5-13]	11.2 [7.2-15]	49 [37-59.5]
Disease type: ext, n (%)	3 (25.0%)	2 (16.7%)	NA
Disease type: pers, n (%)	9 (75.0%)	10 (83.3%)	NA
Female, n (%)	11 (84.6%)	7 (58.3%)	4 (100.0%)
ΑΝΑ, n (%)	12 (92.3%)	10 (83.3%)	NA
NSAID, n (%)	11 (84.6%)	6 ( 50.0%)	NA
DMARD, n (%)	3 (23.1%)	0 (0.0%)	2 (50.0%)
Anti-CCP			
positive, n (%)	0 (0.0%)	0 (0.0%)	1 (25.0%)
negative, n (%)	5 (38.5%)	8 (66.7%)	1 (25.0%)
NA^2^, n (%)	8 (61.5%)	4 (33.3%)	2 (50.0%)
RF			
positive n (%)	0 (0.0%)	0 (0.0%)	1 (25.0%)
negative, n (%)	5 (38.5%)	8 (66.7%)	1 (25.0%)
NA^2^, n (%)	8 (61.5%)	4 (33.3%)	2 (50.0%)

^1^Duration in months

^2^Missing data or sample not taken

*S-Fib* Synovial Fibroblasts, *SF* Synovial Fluid, *RA* Rheumatoid Arthritis

### Synovial fibroblast isolation and culture

For JIA and RA patients, SF was centrifuged at 500 g, 10 min. The cell pellet was washed twice with PBS and seeded in fibroblast-like synoviocytes (FLS) growth medium (Cell Applications) in T-25 flasks (Sarstedt). The SF was centrifuged a second time at 800 g, 10 min and frozen at -80 °C until use. The seeded cells were washed with fresh medium after 24–48 h to remove non-adherent cells, and then passaged at confluency roughly 3 times until S-Fib-like cells were the dominant cell type, in line with previous publications [16, 21–23]. The S-Fib were cryopreserved until use. To confirm their identity, a subset of fibroblasts was characterized using surface markers (CD45 (clone: HI30, PerCP Cy5.5, Biolegend, diluted 1:50), Cadherin-11 (clone: 16G5, PE, Biolegend, diluted 1:50), CD90 (clone: 5E10, APC-Cy7, Biolegend, diluted 1:50) and CD34 (clone: 581, APC, BD, diluted 1:20) and analyzed using flow cytometry (CytoFLEX (Beckman Coulter))). Additionally, healthy adult S-Fib were purchased from Cell Applications and characterized as above.

For co-culture experiments and priming, S-Fib were detached, and 2’500 cells were seeded in 96-well plates (Falcon) in FLS growth medium and cultured for 72 h before use. In some experiments, cells were “primed” with 20% of pooled SF 24 h after seeding, followed by 48 h of culture for a total of 72 h. The 48 h priming period was chosen to limit the effect of different growth kinetics, and based on pilot cytokine stimulation data, showing limited activation of S-Fib at the 24 h mark.

For the generation of S-Fib conditioned supernatants, S-Fib were seeded at 10’000 cells in a 24-well plate (Falcon) in FLS growth medium and primed or not as described above. Next, the cells were washed twice with RPMI-1640 medium, followed by the addition of serum-free RPMI-1640. The cells were cultured for 24 h before harvest of the supernatants.

### Monocyte isolation and co-culture

Heparinized whole blood was collected from healthy adult volunteers upon informed consent. Peripheral blood mononuclear cells (PBMCs) were isolated by density centrifugation (LymphoPrep, Shield-Axis) at 620 g, 20 min, with low break. The PBMC fraction was collected, washed and monocytes were isolated using CD14^+^ magnetic bead separation (Miltenyi) according to the manufacturer’s instructions. The monocyte purity and the number of cells was checked using a hematology analyzer (XN-350, Sysmex). The concentration was adjusted to 1 × 10^6^/mL in RPMI-1640 medium with 10% normal human serum (NHS). S-Fib (cultured as described above) were washed twice with RPMI-1640 medium before the addition of 10^5^ monocytes. Monocytes alone served as control. The cells were cultured for 24 h and analyzed as described below.

### Migration assay

A 24-well plate with 5 μm pore-sized transwell inserts (Corning) was used for the migration assay. HMEC endothelial cells (ATCC) were seeded to the inserts and grown until confluent in MCDB 131 medium (Gibco) supplemented with 10% fetal bovine serum, 10 ng/mL hEGF, non-essential amino acids, sodium pyruvate, penicillin, and streptomycin (PenStrep). The prepared inserts were transferred to a new plate containing oJIA S-Fib supernatants (generated as described above), pooled from 4 different S-Fib donors. Medium alone served as a negative control. Monocytes were isolated as described above, adjusted to 2 × 10^6^/mL in RPMI-1640 and 100 µL was subsequently added to the top of the inserts. The plate was incubated at 37 °C, 5% CO_2_ for 80 min whereafter the inserts were discarded and the cells in the bottom of the wells harvested by gentle pipetting in PBS/1mM EDTA. Monocytes were stained with 1:200 of anti-CD14 (clone: HCD14, BV421, Biolegend) for 20 min, RT. Finally, the cells were washed with PBS, resuspended in 100 µL, and analyzed using flow cytometry (CytoFLEX). The number of CD14^+^ monocytes/µL was used for analysis.

### Monocyte surface marker analysis

Following co-culture with S-Fib as described above, the monocytes were detached using 5mM EDTA/PBS, harvested by centrifugation and stained with anti-CD16 (clone: 3G8, PerCP Cy5.5, BD), anti-MerTK (clone: 590H11G1E3, PE, Biolegend), anti-HLA-DR clone: G46-6 APC-H7, BD) and anti-CD86 (clone: FUN-1, BV650, BD), all diluted 1:200. After a wash with PBS the cells were resuspended and stained with Annexin V (BD) according to the manufacture's instructions. The cells were subsequently analyzed by flow cytometry (CytoFLEX, Beckman Coulter).

### LC-MS/MS

S-Fib were collected, after repeated washing with PBS and detachment using 1mM EDTA in PBS, by centrifugation (300 g for 5 min) and lysed with cold RIPA buffer (Thermo Scientific) supplemented with cOmplete protease inhibitor cocktail (Roche) for 1 h, 4 °C on rotation. The lysates were subsequently stored in -80 °C until use. The cell-lysates were prepared for Mass spectrometry by protein-precipitation using 90% ethanol at -20 °C overnight. The next day, the samples were centrifuged, the supernatant discarded, and the pellet was dried. The protein pellet was dissolved in ammonium bicarbonate (AMBIC) buffer, and reduced with 10 µM dithiothreitol (DTT) for 30 min, 56 °C. Next, alkylation was performed with 20 µM iodoacetamide (IAA) for 30 min, RT in the dark. Protein concentration was measured using a NanoDrop (DS-11 Series Spectrphotometer/Fluormeter, DeNovix). 20 µg of protein was trypsinated (1:50 trypsin: protein ratio) overnight at 37 °C. Finally, trypsin was inhibited with 0.4% trifluoroacetic acid (TFA) and the samples were dried using a SpeedVac and stored at -80 °C until use. The samples were resolved in 20 µl of 2% acetonitrile (ACN) and 0.1% TFA and peptide concentration were determined at 215 nm using NanoDrop. 5 µl of the peptides was injected to Liquid chromatography mass spectrometry (LC-MS).

The samples were analyzed on an Exploris 480 mass spectrometer (Thermo Fischer Scientific) coupled with a Vanquish Neo UHPLC system (Thermo Fischer Scientific). Two-column setup was used on the HPLC system and peptides were loaded into an Acclaim PepMap 100 C18 precolumn (75 μm x 2 cm, Thermo Scientific) and then separated on an EASY spray column (75 μm x 50 cm, C18, 2 μm, 100 Å, ES803A) with the flow rate of 300 nL/min. The column temperature was set at 45 °C. Solvent A (0.1% FA in water) and solvent B (0.1% FA in 80% ACN) were used to create a 120 min non-linear gradient to elute the peptides. For the gradient, the percentage of solvent B was maintained at 5% for 5 min, increased from 5% to 25% for 100 min and then increased to 32% for 12 min and then increased to 45% for 8 min.

The samples were analyzed with a data-dependent acquisition (DDA) in positive mode. The full MS1 resolution were set to 120,000 at m/z 200 and the normalized AGC target was set to 300% with the maximum injection time of 45ms. The full mass range was set 350–1400 m/z. Precursors were isolated with the isolation window of 1.3 m/z and fragmented by HCD with the normalized collision energy of 30. MS2 was detected in the Orbitrap with the resolution of 15,000. The normalized AGC target and the maximum injection time were set to 100% and custom, respectively. The intensity threshold for precursor selection was set to 1e^4^ and custom dynamic exclusion was applied.

The raw DDA data were analyzed with Proteome Discoverer™ 2.5 Software (Thermo Fisher Scientific). Peptides were identified using SEQUEST HT against UniProtKB human database (UP000005640_9606). The search was performed with the following parameters applied: static modification: cysteine carbamidomethylation and dynamic modifications: N-terminal acetylation and methionine oxidation. Precursor tolerance was set to 10 ppm and fragment tolerance was set to 0.02 ppm. Up to 2 missed cleavages were allowed and Percolator was used for peptide validation at a q-value of maximum 0.01. Extracted peptides were used to identify and quantify them by label-free relative quantification. The extracted chromatographic intensities were used to compare peptide abundance across samples. The protein abundances were normalized to the total amount of peptides per sample.

The data was subsequently analyzed in Microsoft Excel and in Rstudio [24]. S-Fib from *n* = 6 donors, with or without priming (total 12 samples), were analyzed. First, proteins with 3 or more unique peptides were selected for further analysis, and contaminants were removed (immunoglobulins, apolipoproteins, hemoglobulin, albumin, and keratins). The data was next log transformed and filtered to be included if the protein was present in 3 or more of the replicates of the untreated S-Fib, as well as in 3 or more of the replicates in the SF-primed S-Fib (thus, it should be present in at least half of the replicates to be included). The proteins fulfilling these criteria were extracted for imputation, using the MissForest algorithm [25].

The imputed data was analyzed using principal component analysis and volcano plots using using paired t-test. The p-values were corrected using Benjamini-Hochberg, and a q-value of below 0.05 was considered significant. The Log2 fold change was calculated, with a cut-off of 1 (to be considered upregulated), or -1 (to be considered downregulated), together with a q-value below 0.05. For pathway enrichment analysis, the differentially regulated proteins were anayzed using Metascape [26] (https://Metascape.org, 2025-09-23) with the whole S-Fib proteome as background. The pathways with a q-value below 0.1 were extracted for visualization.

### T-cell activation

Blood was drawn into heparin tubes from healthy adult volunteers and T-cells were isolated from PBMCs following density centrifugation using the EasySep™ CD4^+^ T-cell isolation kit (Stemcell Technologies) according to the manufacturer’s instructions. The isolated T-cells were stained with 2 µM CellTrace Violet (CTV, Invitrogen) in PBS for 20 min. Next, the T-cells were washed with and resuspended in RPMI-1640 supplemented with 10% fetal calf serum (FCS), 2mM L-glutamine and PenStrep. An uncoated 96-well plate (Falcon) was coated with anti-CD3 (1:1000, Clone: OKT3, Invitrogen) for at least 90 min. 5 × 10^4^ T-cells were seeded in the plate and monocytes, from the co-culture between S-Fib, were added to the T-cells at a 1:20 ratio (monocytes: T-cells) and cultured for 72 h at 37 °C, 5% CO_2_. The monocytes were pooled for each condition (i.e. control, S-Fib and primed S-Fib) and donor and analyzed in duplicate. T-cells without monocytes served as a negative control.

The supernatants for cytokine analysis were collected and frozen in -80 °C until use (described below). For proliferation and surface marker analysis, CTV-stained T-cells were detached through gentle pipetting, washed with PBS, and stained with anti-CD3 (clone UCHT1, alexa fluor 700, 1:200), anti-CD25 (clone M-A251, PerCP Cy5.5, 1:200) and anti-HLA-DR (clone: G46-6 APC-H7, 1:200), all from BD, for 20 min, RT. After a final wash the cells were analysed using flow cytometry (CytoFLEX). T-cells were gated based on CD3 expression.

### Transwell assay

To investigate the impact of direct contact in the monocyte activation process, S-Fib were seeded at 10’000 cells in a 24-well plate (Corning) in FLS growth medium and primed with SF or not as described above. After washing, monocytes (4 × 10^5^) were added to the inserts of 0.4 μm transwells (too small for the monocytes to migrate through) and placed into the wells with S-Fib. The plate was incubated for 24 h and the monocytes, trapped within the inserts, were analysed as above. In addition, supernatants were collected for cytokine analysis as described below.

### Cytokine analysis

IL-1β, IL-6, IL-8 and TNFα in monocyte-S-Fib co-culture supernatants were analyzed using the pro-inflammatory panel 2 (4-plex) according to the manufacturer’s instructions (Mesoscale Diagnostics). Co-culture supernatants were diluted 1:200 and monocultures 1:4. Data was processed, and concentrations were calculated using the Discovery Workbench software (Mesoscale, version 4.0). Supernatants from T-cell culture were analysed for IFNγ and IL-6 using DuoSet ELISA (R&D Systems) according to the manufacturer’s instructions. Supernatants from monocyte-S-Fib cultures in the transwell experiments were pooled for each condition and analyzed for IL-1β, TNFα and IL-6 using ELISA (R&D Systems).

### Statistics

Flow cytometry data was analysed using the CytExpert software (v2.3) or Kaluza (v2.1, both Beckman Coulter). Gating strategies can be found in Supp Fig. 1A-C. Median fluorescence intensity (MFI) values were Log10 transformed before being used. Data is presented as mean with standard deviation if not otherwise stated. Data compared using three groups was analysed using repeated measures-one-way ANOVA or ordinary one-way ANOVA with Tukey’s multiple comparisons test. Data comparing two groups was analysed using paired or unpaired t-test. Mass spectrometry data was analysed as described above. *p* < 0.05 was considered statistically significant. All other data was analysed using GraphPad Prism 10 and Microsoft Excel.

## Results

### Synovial fibroblasts induce comparable monocyte activation regardless of their disease origin

**Fig. 1:**
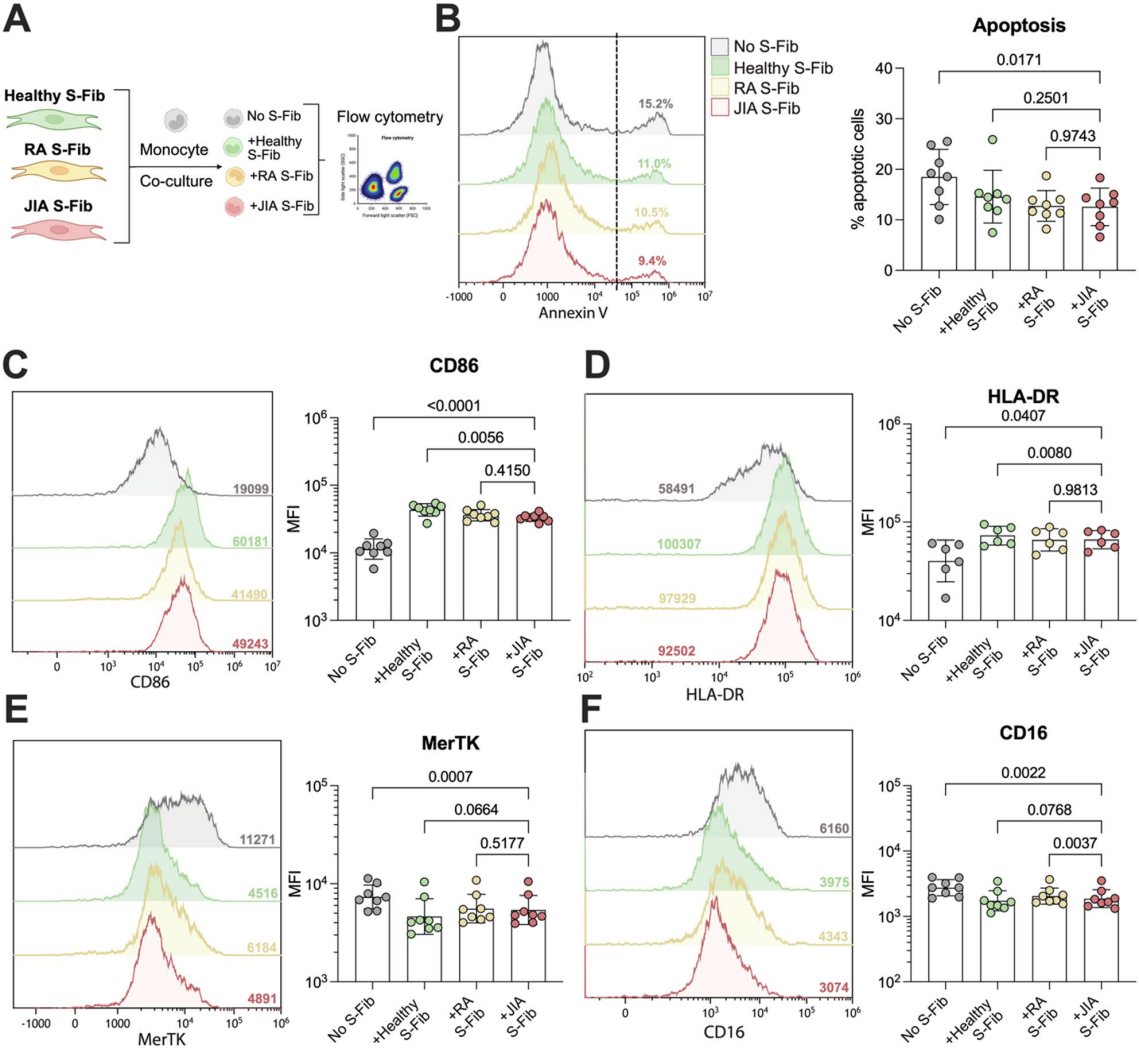
Synovial fibroblasts induce comparable monocyte activation regardless of their origin. (**A**) Synovial fibroblasts (S-Fib) were isolated from healthy controls (*n* = 3), RA patients (*n* = 3–4) or JIA patients (*n* = 5–6) and were co-cultured with healthy monocytes (*n* = 6–8). The monocytes were subsequently analyzed for activation by flow cytometry. (**B**) Displays the degree of apoptosis as measured by Annexin V. Several markers of activation were analyzed, reflecting inflammatory activation: (**C**) CD86 and (**D**) HLA-DR or regulatory activation (**E**) MerTK and (**F**) CD16. Each data point represents a unique monocyte donor, which in turn is the average of co-culture with several S-Fib donors. Data is presented as MFI after log10 transformation, showing mean +/- SD. It was analyzed using repeated measures-one-way ANOVA with Tukey’s multiple comparisons test. *Synovial fibroblasts – S-Fib*, *SF – Synovial fluid*, *MFI – Median fluorescence intensity*

First, we investigated the ability of different synovial fibroblast (S-Fib) states to induce monocyte activation as characterized by surface markers (Fig. 1A). First, we confirmed that co-culture did not result in increased monocyte apoptosis (Fig. 1B). On the contrary, monocytes co-cultured with S-Fib isolated from individuals with oligoarticular juvenile idiopathic arthritis (oJIA) S-Fib had increased viability compared to monocytes alone (*p* = 0.0171). There was no difference compared to monocytes co-cultured with either rheumatoid arthritis (RA) or healthy S-Fib. Moreover, when investigating CD86 and HLA-DR expression, two markers of inflammatory activation, we observed an increase in expression for both CD86 (*p* < 0.0001), and HLA-DR (*p* = 0.0407, Fig. 1C-D) compared to monocytes alone. In addition, the expression in monocytes co-cultured with oJIA S-Fib was comparable to monocytes co-cultured with RA S-Fib, whilst monocytes co-cultured with healthy S-Fib had marginal but statistically increased expression of both markers (*p* = 0.0056 and *p* = 0.0004, respectively). In parallel, CD16 and MerTK, markers of regulatory monocytes, were decreased compared to monocytes alone (*p* = 0.0022 and *p* = 0.0007, respectively, Fig. 1E-F). Monocytes co-cultured with RA S-Fib expressed slightly higher levels of CD16 compared to monocytes co-cultured with oJIA S-Fib (*p* = 0.0037). Taken together, our data indicates that co-culture with S-Fib prolongs monocyte survival induces inflammatory activation defined by cell surface marker expression, with only minor differences between the S-Fib groups.

Next, the identity and phenotype of the S-Fib populations (from oJIA, RA and healthy adults) was confirmed in a subset of samples by comparing the surface marker expression of CD45, cadherin-11, CD90 and CD34. As previously described, fibroblasts isolated this way are CD45^−^CD90^+^ (Supp Fig. 2A). Across oJIA (*n* = 6), RA (*n* = 4), and healthy (*n* = 3) S-Fib, expression of CD90, CD34, and cadherin-11 was largely comparable (Supp. Figure 2B–C), with oJIA S-Fib appearing more similar to RA than to healthy controls. Still, healthy S-Fib seemed to express more of CD90 and CD34 compared to the other groups. Similarly, the S-Fib populations produced comparable levels of IL-6 (Supp Fig. 2D) and IL-8 (Supp Fig. 2E), although RA S-Fib seemed to produce slightly more of both cytokines. In summary, the different S-Fib states in this study evoke a similar monocyte response, although there were some minor differences in the phenotype and cytokine production between the S-Fib populations.

### Synovial fluid induces an inflammatory phenotype in synovial fibroblasts

**Fig. 2:**
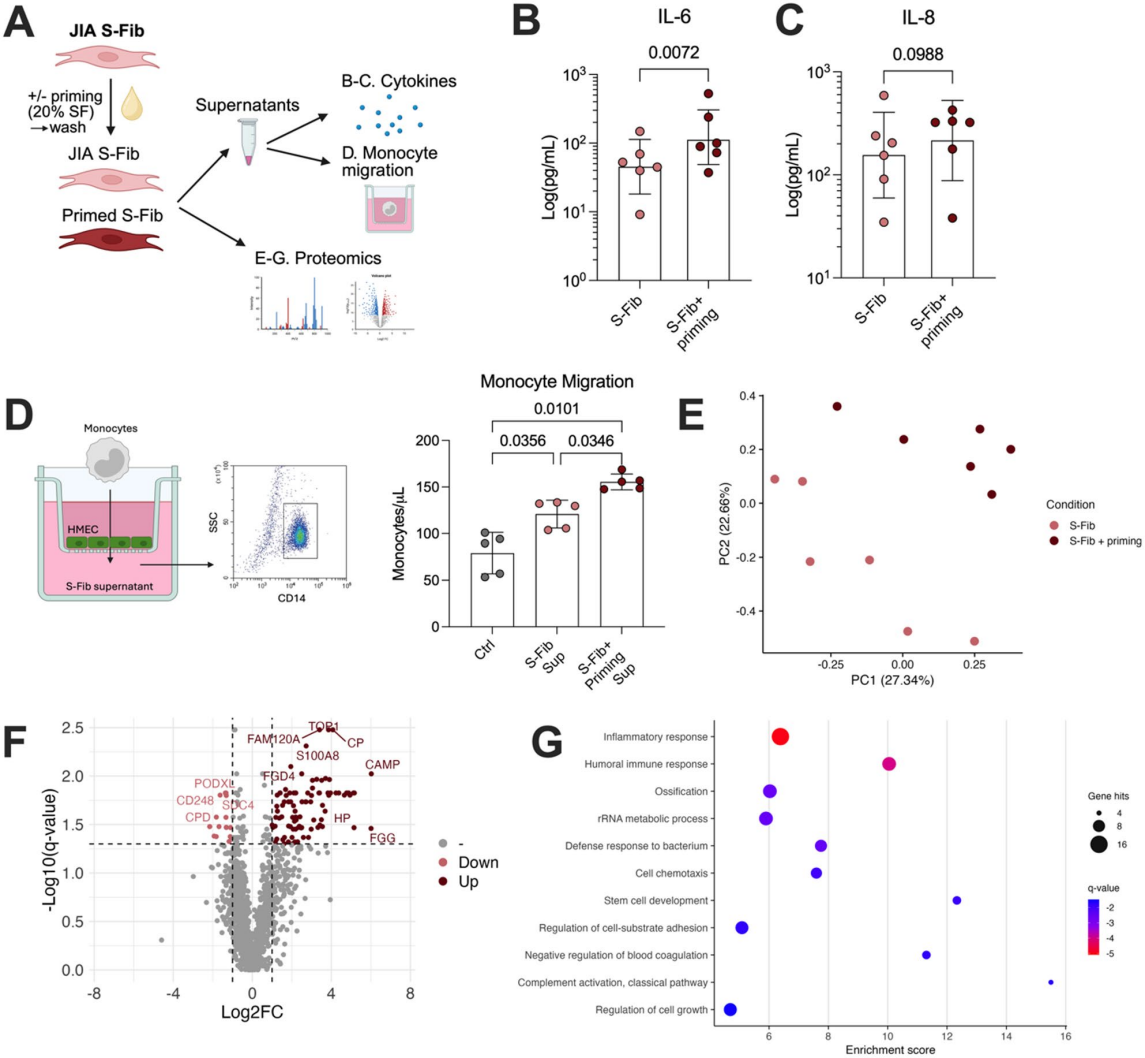
Synovial fluid induces an inflammatory phenotype in synovial fibroblasts. (**A**) Synovial fibroblasts (S-Fib) from oJIA patients were primed or not with a pool of 20% SF, washed and cultured for an additional 24 h before analysis of cytokine production, chemotactic ability, and proteomics. Supernatants of S-Fib cultures from *n* = 6 patients were analyzed for (**B**) IL-6 and (**C**) IL-8 levels. Data is displayed as log10 transformed values. (**D**) Additionally, the supernatants were used to investigate their ability to induce chemotaxis of healthy monocytes in a transwell system seeded with endothelial cells (HMEC). Monocytes (*n* = 5, identified by CD14) were allowed to migrate through the transwells towards a pool of supernatants from *n* = 4 S-Fib donors, before being collected and analyzed by flow cytometry. Moreover, the protein expression of S-Fib were analyzed by LC-MS. (**E**) Shows a principal component analysis of the proteomic differences between SF primed- and control S-Fib (*n* = 6). (**F**) Displays differentially regulated proteins as visualized by a volcano plot. The y-axis displays the FDR adjusted p-values. (**G**) Displays the top enriched processes of the upregulated proteins following pathway analysis. Data is presented as mean +/- SD and was analyzed using paired t-test for two groups or repeated measures-one-way ANOVA with Tukey’s multiple comparisons test for three groups. *S-Fib – Synovial fibroblasts*,* SF – Synovial fluid*,* LC-MS/MS – Liquid-chromatography mass spectrometry*,* JIA – Juvenile idiopathic arthritis*,* FC – Fold change*,* IL – Interleukin*,* HMEC – Human dermal microvascular endothelial cells*,* sup – Supernatant*

S-Fib have been described to lose their positional identity over time in cultures, which might explain the lack of difference in monocyte activation between the S-Fib groups [27]. Thus, we set out to investigate the effect of “re-introducing” S-Fib to the arthritic environment by priming them with a pool of cell-free SF from oJIA patients (Fig. 2A). Given the similar effect across the S-Fib groups in inducing monocyte activation above, we focused on oJIA S-Fib, and the data presented in Fig. 2 was performed only on oJIA S-Fib.

First, we explored the effect of priming on cytokine production (*n* = 6). Priming resulted in increased production of IL-6 (Fig. 2B, *p* = 0.072) but not IL-8 (Fig. 2C, *p* = 0.0988). As expected, a similar effect could be seen for RA S-Fib and healthy S-Fib (Supp Fig. 3A-B). Next, we investigated the ability of S-Fib supernatants to induce monocyte migration in a transwell setting (Fig. 2D). Supernatants from S-Fib as well as primed S-Fib induced monocyte migration (*p* = 0.0356 and *p* = 0.0101, respectively, Fig. 2D) which was more pronounced in primed S-Fib compared to unprimed S-Fib (*p* = 0.0346). At the protein level, investigated by LC-MS/MS of whole cell lysates, there was a pronounced difference between unprimed and primed S-Fib, as visualized by a principal component analysis (PCA, Fig. 2E). In total, 108 proteins were differentially regulated (Fig. 2F). Pathway enrichment analysis revealed that these differentially regulated proteins were involved in processes such as inflammatory responses, cell migration, proliferation and translation (Fig. 2G). Hence, priming of S-Fib with SF from oJIA patients induces an inflammatory phenotype, evidenced by enhanced cytokine production and immune cell chemotaxis.

### Synovial fibroblasts induce pro-inflammatory functional alterations in monocytes, which are potentiated by synovial fluid priming

**Fig. 3:**
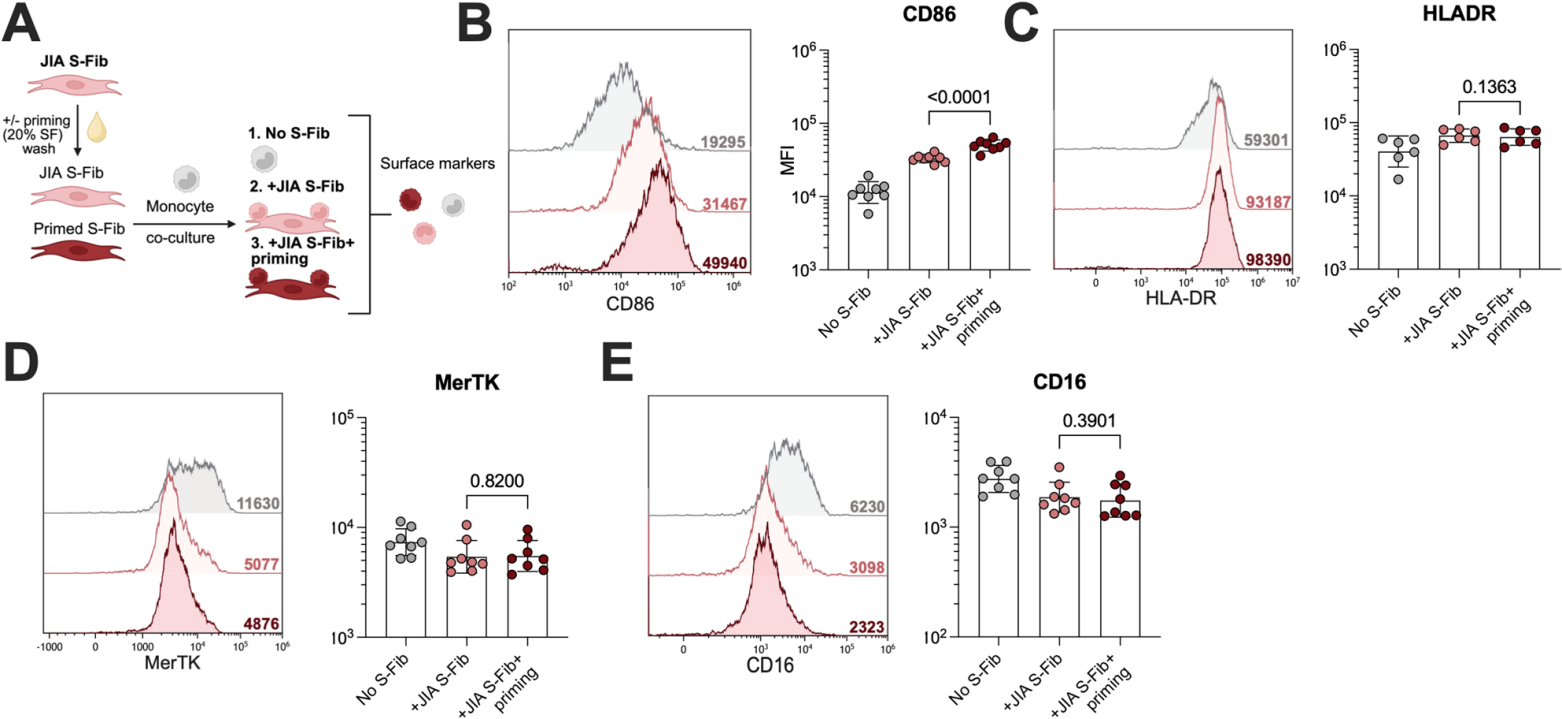
Primed synovial fibroblasts induce increased CD86 expression in monocytes. (**A**) Synovial fibroblasts (S-Fib) were isolated from JIA patients (*n* = 5–6) and primed or not with a pool of 20% SF followed by a wash step and co-culture with healthy monocytes (*n* = 6–8) and monocytes surface marker analysis. The graphs highlight the expression of: (**B**) CD86, (**C**) HLA-DR, (**D**) MerTK and (**E**) CD16. Each data point represents a unique monocyte donor, which in turn is the average of co-culture with several S-Fib donors. Data is presented as MFI following log10 transformation, showing mean +/- SD. Data was analyzed using paired t-test. *Synovial fibroblasts – S-Fib*,* SF – Synovial fluid*,* MFI – Median fluorescence intensity*

**Fig. 4:**
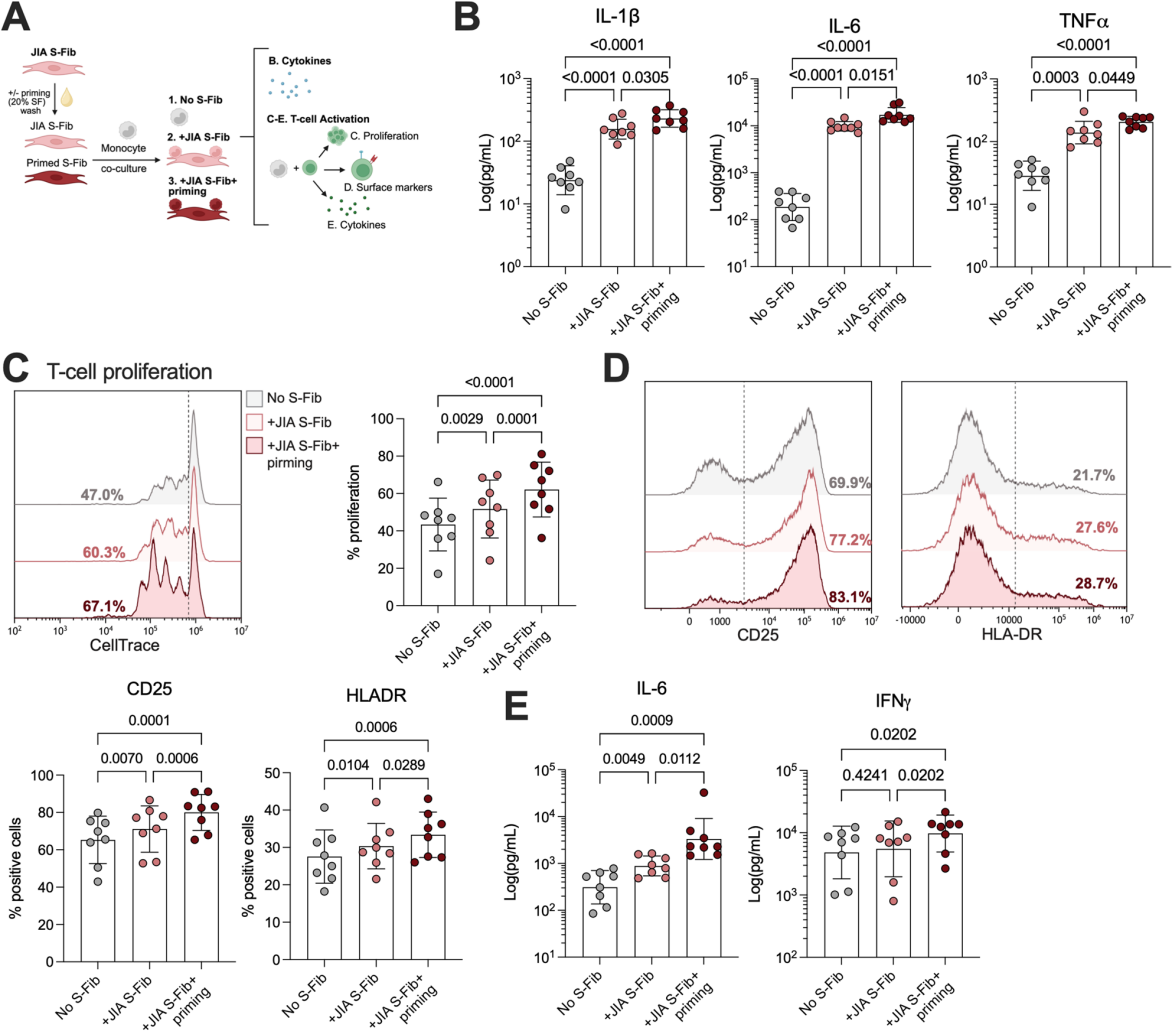
Synovial fibroblasts drive pro-inflammatory monocytes, a process potentiated with prior priming. (**A**) Schematic outline of the analyses performed to investigate monocyte function following co-culture with unprimed or primed synovial fibroblasts (S-Fib). (**B**) Supernatants were analyzed for IL1β, IL-6 and TNFα after 24 h of co-culture. (**C**) Following co-culture, monocytes (*n* = 8) were collected, washed and counted before being incubated with CD3 activated healthy T-cells stained with CellTrace violet (1:20 monocytes: T-cells) for 72 h, which were analyzed for (**C**) proliferation, and (**D**) the expression of CD25 and HLA-DR, by flow cytometry. (**E**) In addition, IL-6 and IFNγ were measured in supernatants following 72 h of monocyte-T-cell co-culture. Each data point represents a unique monocyte donor, which were pooled in equal proportions from co-culture with 5–6 different donors of S-Fib before addition to the T-cells. Data is presented as mean +/- SD and data were analyzed using repeated measures-one-way ANOVA with Tukey’s multiple comparisons test. The cytokine data was log10 transformed before being processesed. *Synovial fibroblasts – S-Fib*,* SF – Synovial fluid*,* IFN – Interferon*,* IL – Interleukin*,* TNF – Tumor necrosis factor*

Next, we studied if co-culture with primed S-Fib induced a different monocyte phenotype compared to unprimed S-Fib (*n* = 6–8, Fig. 3A). Again, we focused on oJIA S-Fib but present data on all S-Fib comparisons in supplementary figures. There was a prominent upregulation of CD86 in monocytes co-cultured with primed S-Fib compared to unprimed S-Fib (*p* = 0.0001, Fig. 3B). However, the expression of HLA-DR, MerTK and CD16 was comparable to that of unprimed S-Fib (Fig. 3C-D). A similar trend was observed in both RA and healthy S-Fib (Supp.Fig 4 A-D).

At the functional level, co-cultures were analyzed for cytokine production and T-cell activation (Fig. 4A). There was a prominent secretion of IL-1β (*p* < 0.0001), IL-6 (*p* < 0.0001) and TNFα (*p* = 0.0003, Fig. 4B) in co-cultures of S-Fib and monocytes compared to monocytes alone. Moreover, co-culturing with primed S-Fib resulted in an even greater secretion of all cytokines compared to unprimed S-Fib (*p* < 0.0449). A similar trend was observed for RA S-Fib and healthy S-Fib (Supp Fig. 5A-C). Next, monocytes were investigated for their ability to induce activation in healthy CD3 stimulated T-cells. Monocytes co-cultured with S-Fib, compared to monocytes alone, induced a greater degree of T-cell proliferation (*p* = 0.0029, Fig. 4C) as well as activation, evidenced by a higher expression of CD25 (*p* = 0.0070) and HLA-DR (*p* = 0.0104, Fig. 4D) as well as IL-6 production (*p* = 0.0049, Fig. 4E) but not IFNγ (*p* = 0.4241). Again, this activation was more pronounced when monocytes were co-cultured with primed S-Fib, were we also observed an increased IFNγ production (*p* = 0.0289). The same trend could be observed for both RA and healthy S-Fib cultures, although monocytes co-cultured with healthy S-Fib were less effective at inducing T-cell activation compared to JIA and RA (Supp Fig. 5D-F). Taken together, our data suggest that monocytes co-cultured with S-Fib acquire an inflammatory phenotype and function, which is more pronounced in monocytes co-cultured with primed S-Fib.

### Cell-cell contact is a crucial mechanism of the fibroblast-monocyte interaction

**Fig. 5:**
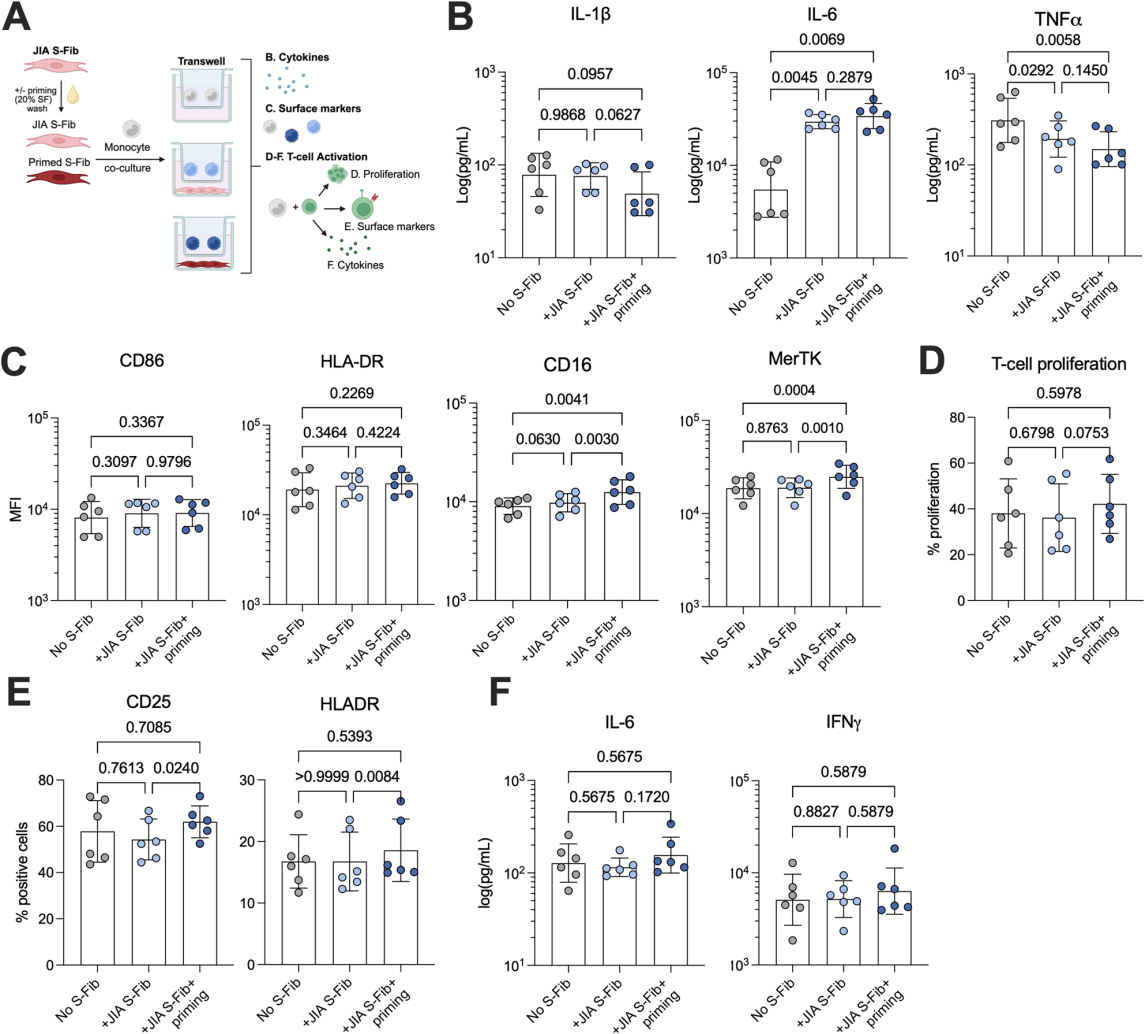
Cell-cell contact is required for inflammatory monocyte activation by synovial fibroblasts. (**A**) Schematic overview of the transwell experiment to investigate the absence of direct contact between monocytes and S-Fib (**B**) Supernatants were analyzed for IL-1β, IL-6 and TNF after 24 h of co-culture (*n* = 6). In parallel, monocytes were detached and analyzed for the expression of (**C**) CD86, HLADR, CD16 and MerTK. Moreover, monocytes (*n* = 6) were subsequently co-cultured with CD3 activated T-cells (1:20 monocytes: T-cells) for 72 h, which were analyzed for (**D**) proliferation, (**E**) the expression of CD25 and HLA-DR, by flow cytometry and (**F**) IL-6 and IFNγ in supernatants. Each data point represents a unique monocyte donor, which were pooled in equal proportions from co-culture with 3 different donors of S-Fib before addition to the T-cells. Data is presented as mean +/- SD and data were analyzed using repeated measures-one-way ANOVA with Tukey’s multiple comparisons test. The cytokine data and MFI data was log10 transformed before being processed. *MFI – Median Fluorescence Intensity*, *Synovial fibroblasts – S-Fib*,* SF – Synovial fluid*,* IFN – Interferon*,* IL – Interleukin*,* TNF – Tumor necrosis factor*

**Fig. 6:**
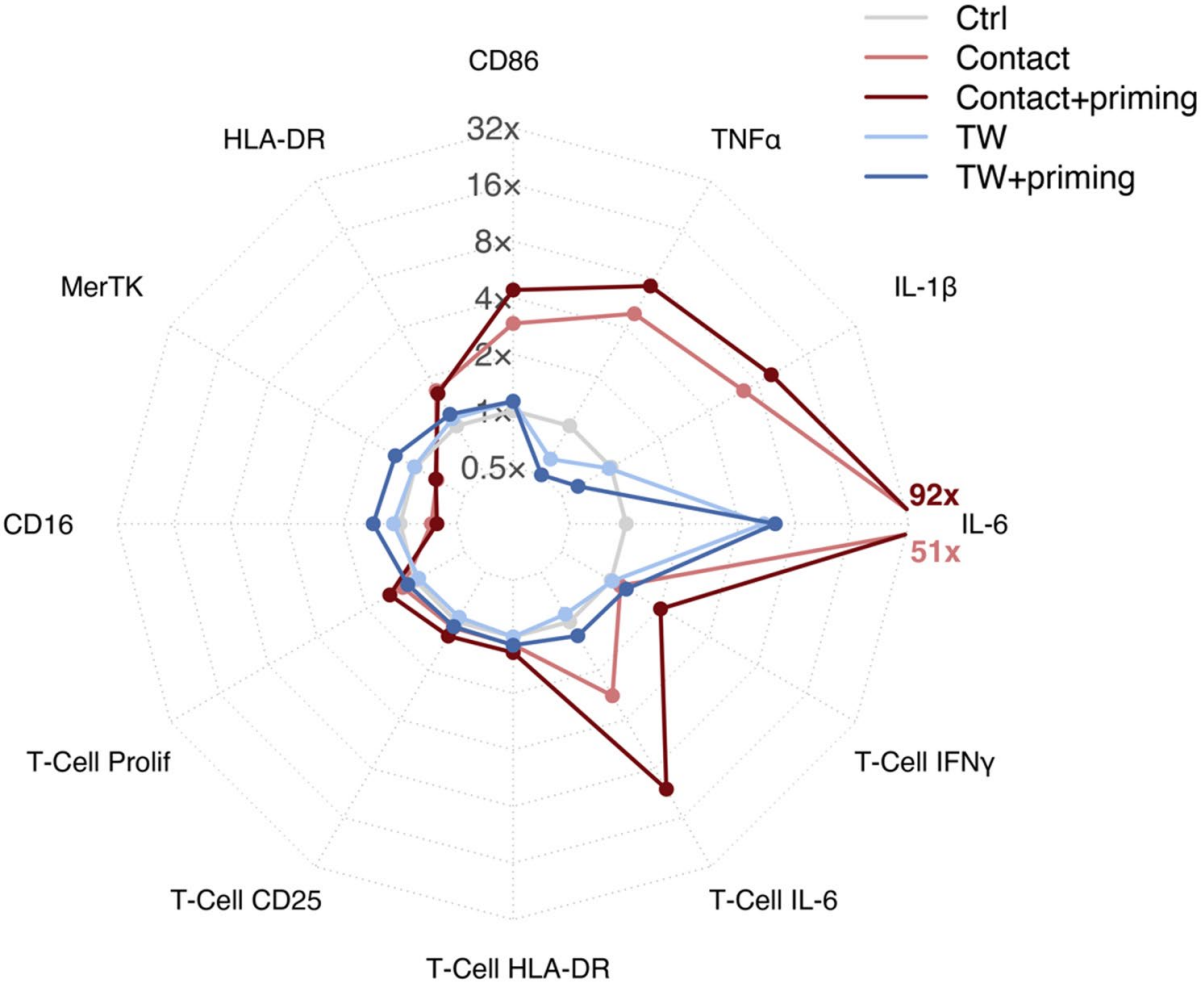
Spider plot outlining a direct comparison of direct contact vs. transwell cultures. A spider plot highlighting the differences in monocytes cultured with synovial fibroblasts (S-Fib) in direct cultures or in transwell cultures. The fold change was calculated for each setting vs. their respective control (monocytes without S-Fib) and presented in a log2 space. For direct contact cultures, the IL-6 levels extended beyond the graph and their fold change is thus marked specifically. *TW - Transwell*,* IFN – Interferon*,* IL – Interleukin*,* TNF – Tumor necrosis factor*,* Prolif - Proliferation*

To investigate whether the effect of S-Fib on monocytes is contact-dependent, we cultured monocytes in 0.4 μm transwells, allowing for exchange of soluble factors but not direct cell-contact as the pores are too small (Fig. 5A, *n* = 6). We did not observe the same pattern of activation compared to direct co-culture. Firstly, as with direct contact, there was an increase in IL-6 production in monocyte-S-Fib transwell cultures (*p* < 0.0069), although there was no difference in IL-6 between primed and unprimed S-Fib (*p* = 0.2879, Fig. 5B). However, in contrast to direct contact cultures, there was no increase in IL-1β or TNFα, on the contrary, their levels were lower in transwell cultures with S-Fib as opposed to monocytes alone (Fig. 5B). Secondly, at the monocyte surface level, there was no upregulation of CD86 or HLA-DR in monocytes cultured in transwells with S-Fib compared to monocytes alone (Fig. 5C). However, in contrast to monocytes cultured with S-Fib in direct contact, there was an upregulation of MerTK (*p* < 0.001) and CD16 (*p* < 0.0041) expression in monocytes cultured with primed S-Fib, compared to both monocytes alone and unprimed S-Fib, in a transwell system (Fig. 5C). Thirdly, at the T-cell level, there was no difference in the ability of the monocytes to induce T-cell proliferation, although there was a trend of increased proliferation in primed vs. unprimed S-Fib transwell cultures (*p* = 0.0753, Fig. 5D). Similarly, when comparing primed vs. unprimed S-Fib cultures, there was an upregulation of CD25 (*p* = 0.0240) and HLA-DR (*p* = 0.0084, Fig. 5E). Finally, at the cytokine level, there was no difference in production of either IFNγ or IL-6 (Fig. 5F). Hence, our data suggest that there is some activation induced by soluble factors, but cell-cell contact is a crucial mechanism in mediating the increased inflammatory phenotype in monocytes induced by S-Fib. To summarize our data and make a visual comparison between direct contact and transwell cultures, a summary figure based on fold change data can be found in Fig. 6.

## Discussion

Monocytes are key effector cells in the pathogenesis of arthritis, but little is known of the crosstalk between the synovial fibroblasts (S-Fib) and monocytes, especially in JIA. Here, we explored how S-Fib from patients with JIA, rheumatoid arthritis (RA) or healthy adults induced activation of healthy monocytes. Regardless of the source of the S-Fib, they induced inflammatory activation of monocytes, evidenced by surface marker expression, cytokine production and enhanced T-cell activation capabilities. Moreover, priming the S-Fib with synovial fluid (SF) from patients with oJIA, mimicking a disease relapse, resulted in an enhanced cytokine production and monocyte recruitment. Accordingly, primed S-Fib induced an even greater inflammatory phenotype in monocytes compared to unprimed S-Fib, suggesting that S-Fib interplay with the local environment to potentiate the inflammatory response in monocytes. These features were dependent on direct cell-cell contact. Taken together, these data suggest a crucial role for S-Fib in driving inflammation by promoting inflammatory monocytes, thus supporting the notion of targeting cell-cell interactions in the treatment of arthritis.

Monocytes are prevalent in the inflamed joint in JIA, where they display signs of activation and functional alterations [4, 6, 28]. Synovial monocytes express high levels of co-stimulatory molecules, such as CD40 and CD86 [6]. They are also prominent cytokine producers, induce T-cell proliferation and display epigenetic alterations, indicative of an activated phenotype [4, 29]. However, few studies have investigated how the monocytes become activated. Importantly, we have previously shown that the environment, SF, induces the regulatory aspects observed in synovial monocytes, but the inflammatory aspects are less clear. Interestingly, S-Fib are gaining increased attention as drivers of inflammation [30]. We have recently shown that co-culture with healthy adult S-Fib induces an inflammatory phenotype in monocytes [5]. Here, we observed a similar trend using S-Fib from oJIA or RA patients. The S-Fib induced CD86 and HLA-DR expression, cytokine production and an increased ability to induce T-cell activation in healthy monocytes. T-cells are well described in the pathogenesis of arthritis [31, 32]. Hence, the induction of T-cell activation is likely an unfavorable effect of the monocytes in this environment, potentially supporting breach of tolerance and an adaptive immune response. Moreover, reduction in the clearance markers CD16 and MerTK suggests that the monocytes may be less efficient at clearing, potentially resulting in accumulation of immune complexes and debris.

Notably, we observed only minor differences between S-Fib isolated from healthy and arthritic joints, suggesting a general mechanism of S-Fib in driving inflammatory monocytes in vitro. This might be explained by S-Fib losing their positional identity over time in culture, which could influence their function, as they converge to a similar phenotype [27]. It is likely that, given that S-Fib from different regions of the synovial membrane have different functions [8], if S-Fib are isolated from tissue it would be feasible to do experiments in early passages to study these differences. However, this is ethically and logistically challenging in pediatric patients, and several studies use SF isolated S-Fib [16, 21, 33, 34]. Another explanation could be that basic contact between these cells is meant to induce activation, as the healthy synovium is generally devoid of monocytes in steady state. Regardless, these data support a role of S-Fib in driving activation of monocytes, reminiscent of some features observed in patients’ synovial monocytes [5].

In addition, there are limited studies on the effect of re-introducing S-Fib into their inflamed environment by stimulating them with SF. Stimulation of S-Fib with cytokines found in inflamed SF, such as IL-1β and TNFα, induces cytokine production, and a recent study using inflamed SF made a similar observation [11, 18]. In addition, studies in animal models have shown that repeated stimulation of S-Fib results in a more pronounced phenotype, driven by metabolic alterations and intracellular C3 signaling [10]. These S-Fib are, in turn, more invasive and migratory. Therefore, it is suggested that repeated stimulation of S-Fib drives a sustained, possibly chronic, inflammatory response [10, 35, 36]. Interestingly, S-Fib, but not dermal fibroblasts, are sensitive to priming, mounting a stronger inflammatory response upon re-activation [35]. Here, we added to these studies as SF priming induced cytokine production and enrichment of processes related to inflammation, migration and translation in the S-Fib. Importantly, we also found that supernatants of the S-Fib promote migration of monocytes. In addition, we have previously observed that S-Fib induce migration of healthy neutrophils, but not activation [37]. Interestingly, this suggests that S-Fib may have diverse interactions with different immune cells. Indeed, SF priming of the S-Fib potentiated them to drive a more pronounced inflammatory phenotype in the monocytes. Specifically, co-culture with SF-primed S-Fib promoted monocytes to induce more T-cell activation, express more CD86 and to produce more cytokines, compared to co-culture with S-Fib without priming. Therefore, we highlight another mechanism of how S-Fib priming amplifies the inflammatory monocyte phenotype which could be a key pathogenic driver of persistent or more severe disease. Importantly, as discussed above these observations are in contrast to the effect of inflamed SF on monocytes alone, which we previously showed induces a regulatory phenotype with reduced ability to induce T-cell activation [5].

We found that cell-cell contact is required for full induction of the monocyte phenotype by S-Fib. Although, soluble factors seemed to induce some alternative activation in the transwell system (devoid of direct cell contact). In the transwell system, we observed upregulation of CD16, MerTK, IL-6 production and some limited degree of T-cell activation (when comparing primed vs. unprimed S-Fib cultures). The presence of S-Fib alone does not explain the increased IL-6 production since it was still strikingly higher in transwell cultures (Fig. 5B) compared to S-Fib alone (Fig. 2B). Therefore, it is likely that a soluble exchange occurs between S-Fib and monocytes, triggering IL-6 production by one or both of these cell types. Regardless, the observed upregulation of CD16 and MerTK on the monocytes could potentially be attributed to cytokines such as IL-6. Indeed, IL-6 has previously been shown to, at least, induce CD16 expression [5]. However, given that there was no difference in CD16 and MerTK expression between monocytes-S-Fib cultures and monocytes alone, there is likely other factors influencing the expression too. Moreover, monocytes cultured in transwells with primed S-Fib seemed to induce some degree of T-cell activation compared to unprimed S-Fib, as evidenced by CD25 and HLA-DR upregulation. Yet, this did not reach significance compared to control monocytes cultured without S-Fib, making the relevance of these findings hard to interpret.

On the other hand, there was no upregulation of CD86 or HLA-DR in monocytes in transwell cultures with S-Fib. In addition, there was no increase in IL-1β or TNFα production, on the contrary, there was less production in monocytes-S-Fib transwell cultures compared to monocytes alone. Finally, there was no difference in T-cell proliferation or cytokine production, suggesting limited T-cell activation in this system. Taken together, our data suggests that soluble exchange between monocytes and S-Fib influence the monocytes to some degree. However, direct cell-cell is required for full activation. Even though, to our knowledge, no previous study has investigated SF-primed S-Fib, one study observed increased attachment to activated S-Fib by immune cells, which was attributed to CD106 (VCAM-1) [34]. Therefore, the precise mechanisms of how S-Fib mediate monocyte activation remains to be determined. Likely candidates include adhesion molecules, such as VCAM-1 and ICAM-1. This mechanism needs to be explored in a future study.

This study has some limitations. A few of our assays, primarily the analysis of cytokines, cannot discriminate if the source of the cytokines is the S-Fib or the monocyte. Yet, the most prevalent cell in these cultures is the monocyte. Regardless, we measure a substantial increase in cytokine production in co-cultures, suggesting that interaction between these cells drive production. We were not able to collect biopsies to isolate S-Fib from patients due to ethical and logistical reasons, which would arguably be a more relevant source of S-Fib. Additionally, it is important to note that the healthy S-Fib were isolated from the tissue, hence a direct comparison should be interpreted carefully. Indeed, RA S-Fib seemed more alike to JIA S-Fib, and the difference to healthy S-Fib could likely be due to the isolation method. Moreover, the cells were cryopreserved before use, which could have an impact on their function and properties. However, all cells were handled in a similar fashion to limit this. Furthermore, we did not have access to healthy SF for priming purposes. Nonetheless, others have described how common inflammatory mediators drive activation in S-Fib, highlighting that they respond to various factors found in inflamed SF [11]. Finally, some patients donating S-Fib were treated with disease-modifying anti-rheumatic drugs (DMARDs) at the time of sampling. These patients were included to increase the number of replicates and ensure more robust results. Yet, DMARDs could have an impact on the phenotype and function of the S-Fib. Although, given the similarities across the different patient groups, the influence of DMARDs is likely minor. Additionally, given that the cells are isolated through passaging, we believe that drug remnants and their influence would be minor although permanent phenotypic or epigenetic changes due to the drugs cannot be ruled out.

## Conclusions

In conclusion, we show that S-Fib drive inflammatory monocyte activation in a contact dependent manner, a process potentiated by prior priming of the S-Fib with SF and thus emphasize that cell-cell interactions could be a viable option to explore for novel treatment strategies in arthritis.

## Supplementary Information

Supplementary Material 1.

## Data Availability

The raw proteomics data have been deposited to the ProteomeXchange with the dataset identifier PXD041459. The other data is available from the corresponding author upon reasonable request.

## Abbreviations

S-Fib: Synovial Fibroblasts
SF: Synovial Fluid
oJIA: Oligoarticular Juvenile Idiopathic Arthritis
RA: Rheumatoid Arthritis
