# Supplementary material for: Synovial fluid potentiates local fibroblasts to drive monocyte activation in juvenile idiopathic arthritis
Source: Arthritis Res Ther. 2026 Feb 28;28:81. doi: 10.1186/s13075-026-03776-z (PMC13059252; doi:10.1186/s13075-026-03776-z)
Supplement: Supplementary file 1 — Supplementary Material 1. [file 13075_2026_3776_MOESM1_ESM.docx]

**Supplementary File**

**Synovial fluid potentiates local fibroblasts to drive monocyte activation in juvenile idiopathic arthritis**

Tobias Schmidt^1,2,3*^, Anki Mossberg, Ph.D.^1,3^, Petra Król, M.D., Ph.D. ^4^, Meliha C Kapetanovic, MD, Ph.D.^5^, Jon T Einarsson, M.D, Ph.D. ^5^, Adam P Croft M.D., Ph.D.^6 -8^, Anders A Bengtsson M.D., Ph.D.^5^, Fredrik Kahn M.D., Ph.D.^3,9^ and Robin Kahn M.D., Ph.D.^3,4^

**Author affiliations**

^1^Department of Pediatrics, ^2^Rheumatology, Clinical Sciences Lund, Lund University, Sweden.

^3^Wallenberg Center for Molecular Medicine, Lund University, Sweden.

^4^Lund University, Skane University Hospital, Department of Clinical Sciences Lund, Pediatrics, ^5^Rheumatology, ^9^Infectious disease, Lund, Sweden

^6^Department of Inflammation and Ageing, ^7^Research into Inflammatory Arthritis Centre Versus Arthritis (RACE), University of Birmingham, Birmingham B15 2TT, UK.

^8^National Institute for Health and Care Research (NIHR) Birmingham Biomedical Research Centre, Birmingham B15 2TH, UK.

*Corresponding author: Tobias Schmidt, tobias.schmidt@med.lu.se, +46 46 222 17 66 Address: BMC B14, Department of Rheumatology, Klinikgatan 26, 22185 Lund, Sweden.


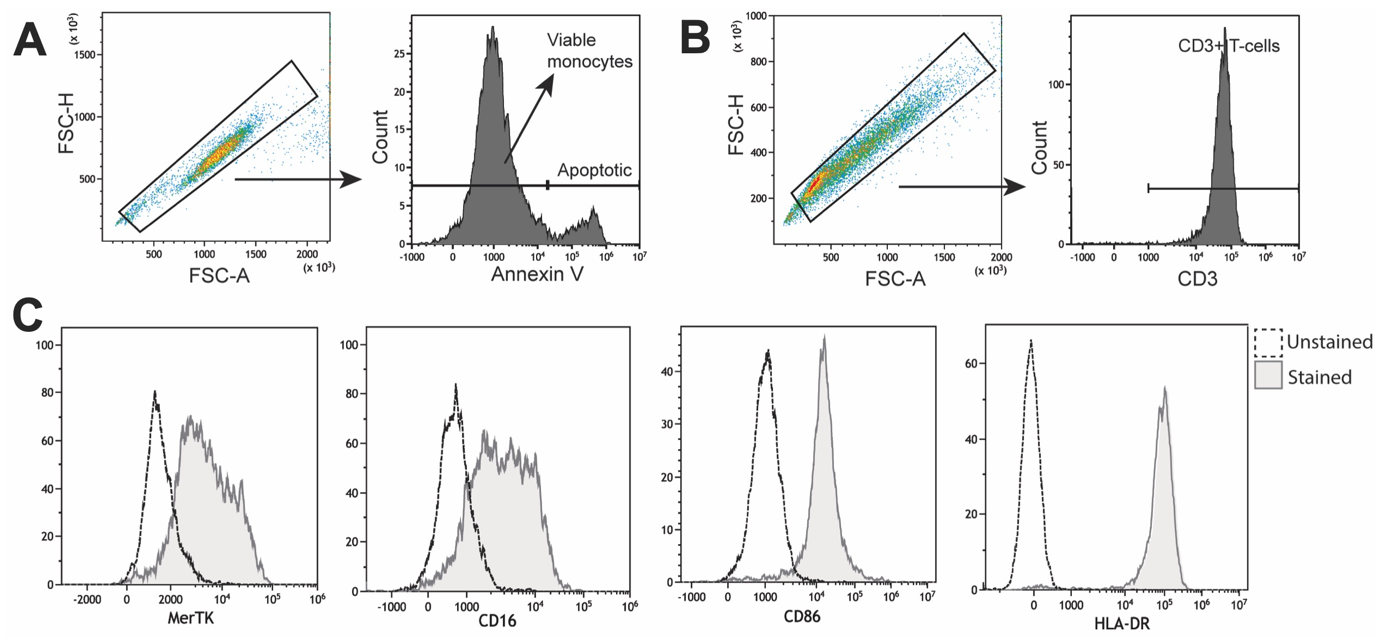
**Supplementary Figure 1. Gating strategy** (**A**) Shows the gating strategy for monocytes and (**B**) for T-cells. (**C**) Displays the expression of the analyzed monocyte markers with an unstained control following gating according to panel A. *FSC – forward scatter.*


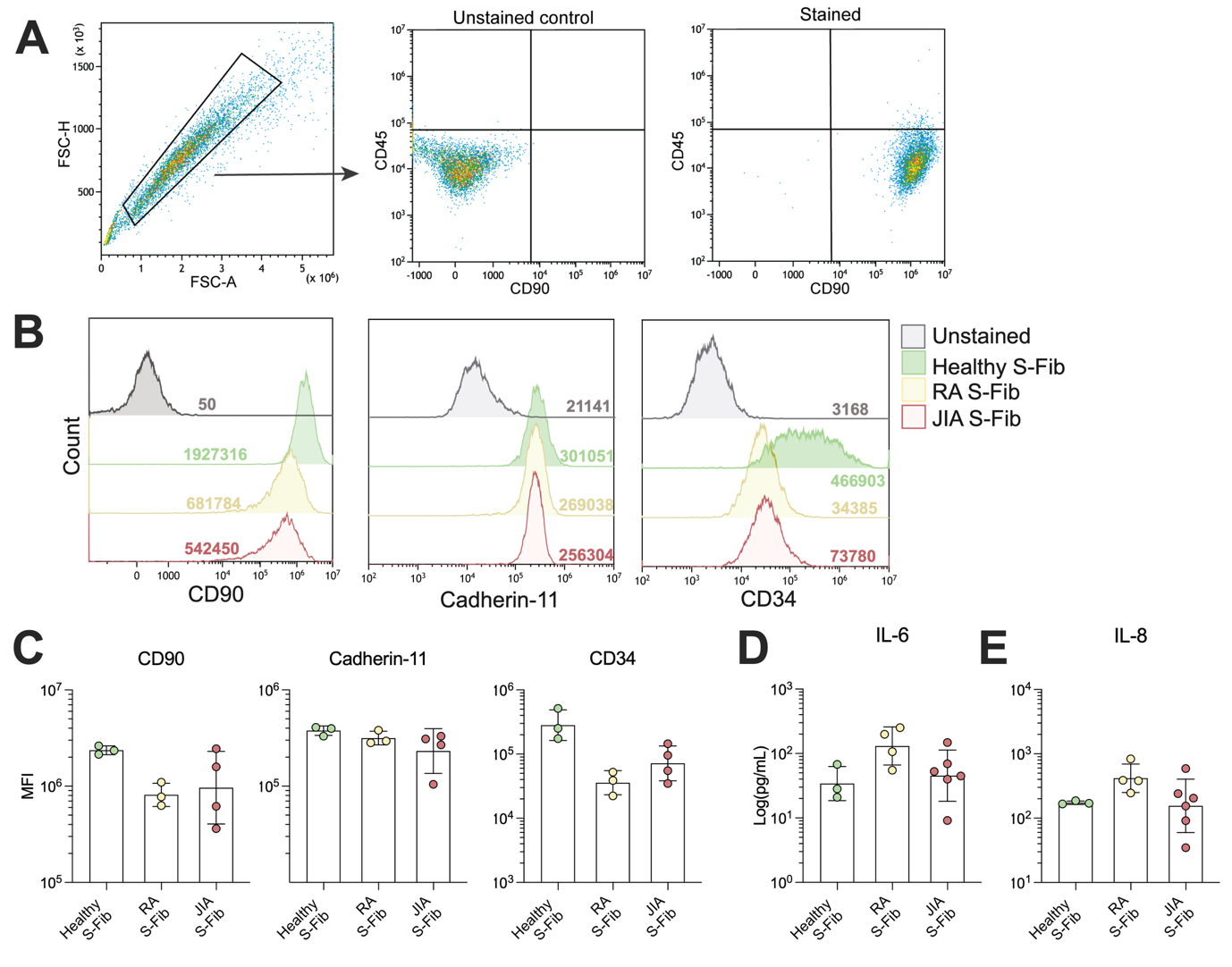


**Supplementary Figure 2**. Characterization of synovial fibroblasts (**A**) Shows the gating strategy for synovial fibroblasts (S-Fib), defined as CD45 negative and CD90 positive. (**B**) Displays representative histograms of the three markers investigated and (**C**) shows them quantified in n = 3 Healthy S-Fib, n = 4 RA and n = 6 JIA. S-Fib. (**D**) Indicates cytokine production of IL-6 and (**E**) IL-8 as measured by Mesoscale. Data is displayed as mean +/- SD. Data was log10 transformed before being processed. *FSC – forward scatter, MFI – Median fluorescence intensity.*


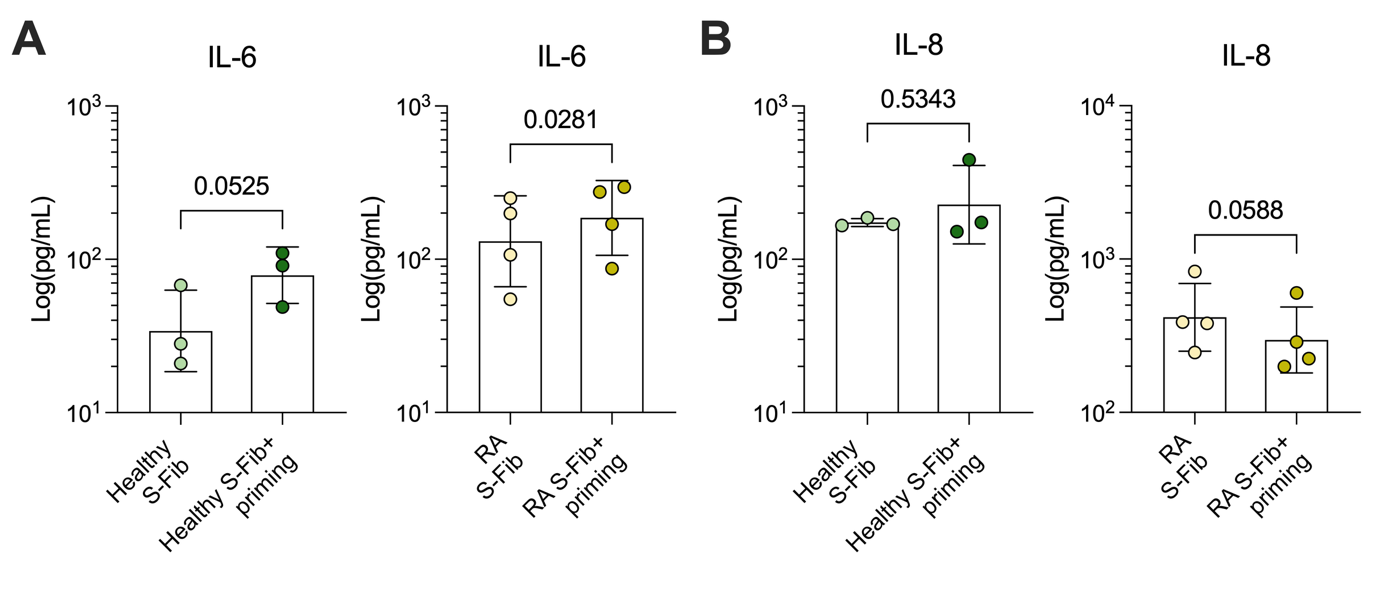
**Supplementary Figure 3. Cytokine production in synovial fibroblasts from RA or healthy controls.** Synovial fibroblasts were primed or not with 20% synovial fluid for 48 h before being washed and cultured for an additional 24 h. (**A**) Shows IL-6 levels or (**B**) IL-8. Bars show mean +/- SD following log10 transformation and statistical comparisons were made using paired t-test. *S-Fib – Synovial Fibroblasts*


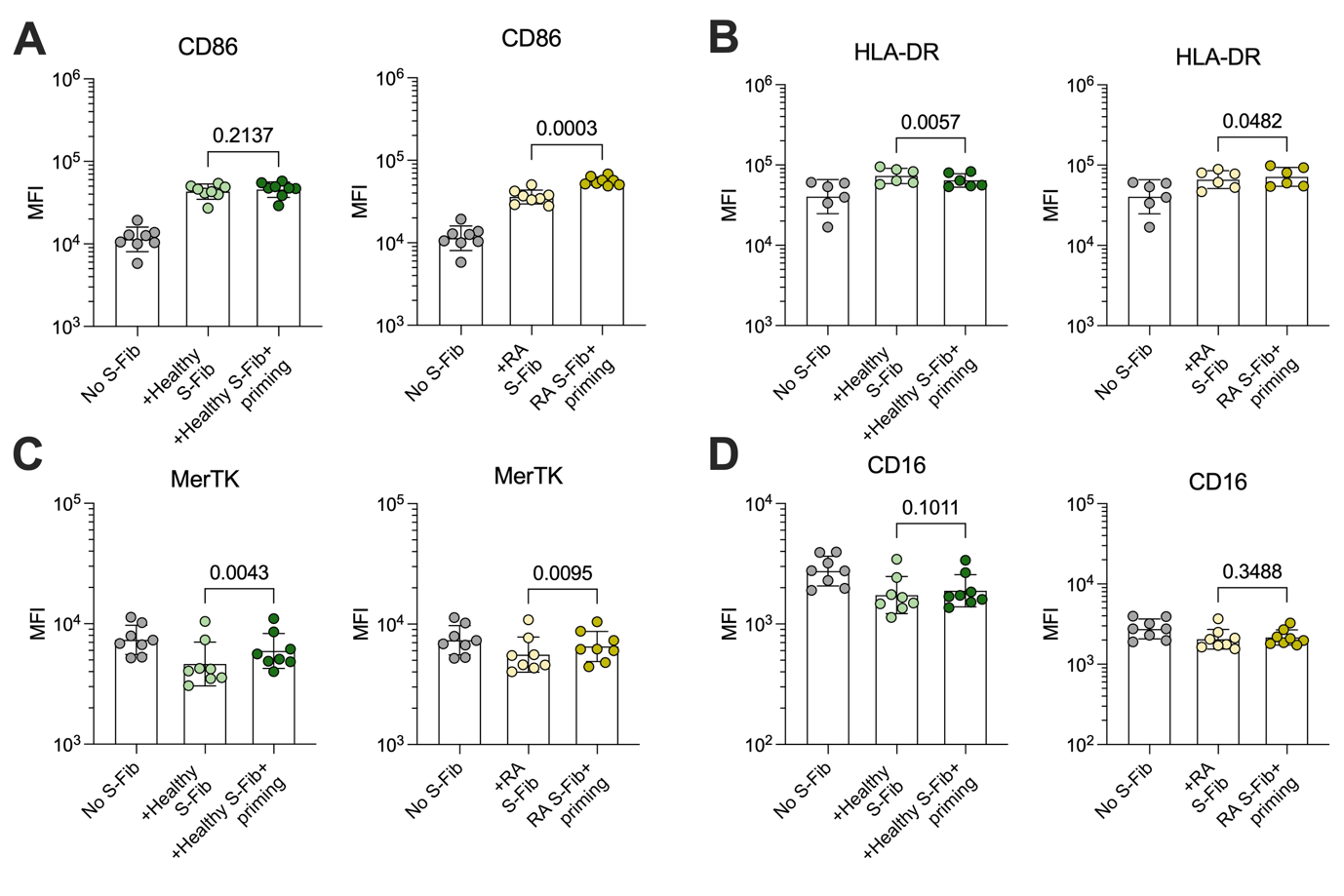


**Supplementary Figure 4. Priming of healthy or RA synovial fibroblasts induce similar activation as JIA synovial fibroblasts** (**A**) Synovial fibroblasts (S-Fib) were isolated from healthy donors (n = 3) RA patients (n = 3-4) and primed or not with a pool of 20% SF followed by co-culture with healthy monocytes (n = 8) and monocytes surface marker analysis. The graphs highlight the expression of: (**A**) CD86, (**B**) HLA-DR, (**C**) MerTK and (**D**) CD16. Each data point represents a unique monocyte donor, which in turn is the average of co-culture with several S-Fib donors. Data is presented as MFI following log10 transformation and bars show mean +/- SD. The data was analyzed using paired t-test. *Synovial fibroblasts – S-Fib, SF – Synovial fluid, MFI – Median fluorescence intensity.*


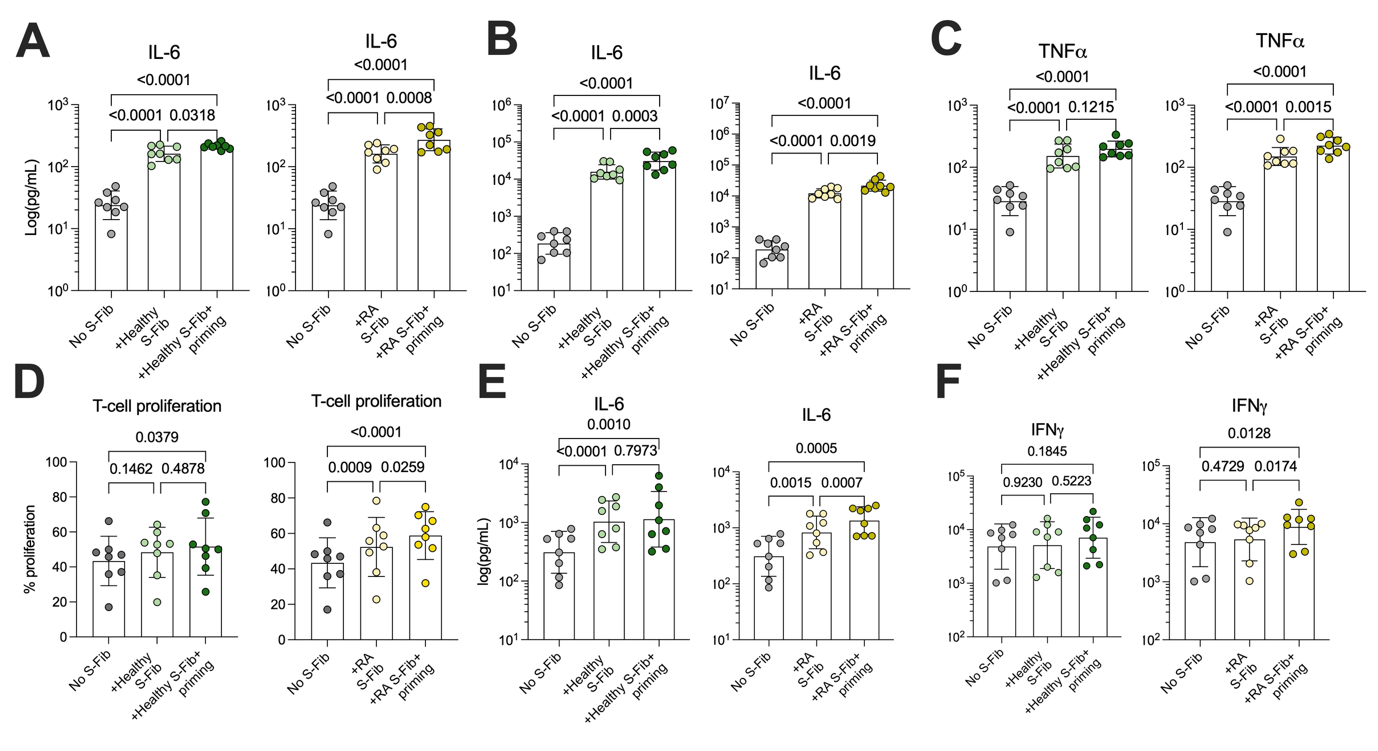


**Supplementary Figure 5. Synovial fibroblasts with or without priming from RA patients or healthy controls induce pro-inflammatory monocytes.** S-Fib from RA or healthy controls were primed or not with SF. Supernatants were analyzed for (**A**) IL1β, (**B**) IL-6 and (**C**) TNFα after 24 h of co-culture with healthy monocytes. (**C**) Following co-culture, monocytes (n = 8) were incubated with CD3 activated healthy T-cells stained with CellTrace violet (1:20 monocytes:T-cells) for 72 h, which were analyzed for (**D**) proliferation, and (**E-F**) IL-6 and IFNγ in supernatants following 72 h of monocyte-T-cell co-culture. Each data point represents a unique monocyte donor, which were pooled in equal proportions from co-culture with 3 (healthy S-Fib) or 3-4 (RA S-Fib) different donors of S-Fib before addition to the T-cells. Data is presented as mean +/- SD and data in three groups were analyzed using repeated measures-one-way ANOVA with Tukey’s multiple comparisons test. The cytokine data was log10 transformed before being processed. *Synovial fibroblasts – S-Fib, SF – Synovial fluid, IFN – Interferon, IL – Interleukin, TNF – Tumor necrosis factor.*
